# Nutritional Renaissance and Public Health Policy

**DOI:** 10.18314/jnb.v3i1.145

**Published:** 2017-08-25

**Authors:** TC Campbell

**Affiliations:** Division of Nutritional Sciences, Cornell University, NY 14851, USA

**Keywords:** Nutrition definition, Pleiotropy, Epitropy, Whole plant-based foods, Animal protein, Chronic disease, Antioxidants, U.S. Affordable Care Act, U.S. Dietary Guidelines

## Abstract

The science of nutrition has long been entrapped in reductionist interpretation of details, a source of great confusion. However, if nutrition is defined as the integration of countless nutrient factors, metabolic reactions and outcomes, biologically orchestrated as in symphony, its relevance for personal and public health would be less confusing and more productive. This more wholistic interpretation may be observed at the cellular and physiological levels and may be described, in part, by the concept of pleiotropy (multiple cell-based effects from one nutrient source), together with its more expansive cousin, epitropy (multiple cell-based effects from multiple nutrients). There are many consequences. First, wholistic interpretation helps to explain the profound but little-known health benefits of whole plant-based foods (not vegan or vegetarian) when compared with whole animal-based foods and/or with the nutritionally variable convenience foods (generally high in fat, salt, refined carbohydrates and low in complex carbohydrates). Second, wholistic interpretation explains why the U.S. Dietary Guidelines and related public policies, which are primarily conceived from reductionist reasoning, serve political agendas so effectively. If diet and health advisories were to acknowledge the biological complexity of nutrition, then make greater use of deductive (top down) instead of inductive (bottom up) reasoning, there would be less confusion. Third, wholistic nutrition, if acknowledged, could greatly help to resolve the highly-polarized, virtually intractable political debate on health care. And fourth, this definition tells why nutrition is rarely if ever offered in medical school training, is not one of the 130 or so medical specialties, and does not have a dedicated research institute at U.S. National Institutes of Health. Nutrition is a wholistic science whereas medical practice is reductionist, a serious mismatch that causes biased judgement of nutrition. But this dichotomy would not exist if the medical practice profession were to understand and adopt wholistic interpretation. Reductionist research, however, is crucially important because its findings provide the granular structure for wholistic interpretation—these two philosophies are inescapably interdependent. Evidence obtained in this manner lends strong support to the suggestion that nutrition is more efficacious and far more affordable in maintaining and restoring (treating) health than all the pills and procedures combined. Admittedly, this is a challenging paradigm for the domain of medical science itself.

## Introduction

Nutrition is a word often spoken but little understood. It deserves a fresh, new definition. In its simplest form, nutrition is the biological process by which food creates, maintains and restores health. The word ‘diet’, which is not the same as ‘nutrition’, is the array of foods customarily eaten. Diets vary, and according to one of the more informative perspectives, from herbivorous to carnivorous practices, often involving addictive excursions into sugar, fat and salt laden foods, uncooked foods, low carb/high protein foods and a variety of practices in between.

It has long been known that food has an important effect on health, ever since Hippocrates said that food is our medicine. Twenty-four hundred years later, however, there still are sharp differences of opinion as to which foods optimize health and prevent chronic diseases like cardiovascular disease, diabetes, obesity and cancer, among others. One of the more telling observations on the association of food with human health shows that chronic disease rates change when dietary practices change over time and/or when people migrate to new lands and adopt new dietary practices. Mainly, these changes in disease rates occur because of nutritional practices rather than genetic predisposition. (1–3) Disease rates generally increase with the consumption of more animal-based and refined, processed foods and less whole plant-based foods. These high-risk diets are higher in calories, fat, protein and refined carbohydrates and lower in antioxidants and complex carbohydrates.

## A Focus on Individual Nutrients

For more than a century, experimental investigations of nutrition have typically focused on the activities and quantities of individual nutrients, often experimentally investigated in isolation. Whole foods and their products are judged by their individual nutrient contents. Nutrient requirements and recommended allowances refer to specific nutrients to be consumed and it is these amounts that provide the basis for recommendations on the use of whole foods.

Focusing on individual nutrients to explain the health value of food is a profound belief system, hardly ever questioned. In recent years, one helpful step in a new direction is the use of dietary patterns to better understand food and nutrient associations with outcomes. It is a welcome development but, in my reading of this rapidly expanding literature, patterns of food and nutrient consumption is still being interpreted as if nutrients biologically function independently, perhaps even additively [4], even within their food context. Generally described as reductionism, this research has produced a vast amount of information but how well this strategy advances a broader understanding of nutrition at the tissue level and on overall public health leaves many unanswered questions, as follows.

- how well does nutrient intake (dose) predict nutrient function (response) at the tissue level?
- do nutrients consumed in pill form provide the same benefits as nutrients consumed in food form?
- for a single nutrient, how much of its functional levels in tissue are affected by other nutrients?
- how can ever changing rates of nutrient transfer of, say, 5–25% variation, through each of the stages of digestion, intestinal absorption, intravascular active/inactive modes of transport, tissue disposition, intracellular metabolism, storage and excretion be factored into estimates of the association of nutrient intake with tissue nutrient response?
- can yet-to-be-discovered nutrient-nutrient interactions be chemically predicted and biologically interpreted?
- how can the clinical measurement of the storage form of a nutrient be meaningful when one of its functional metabolites may be 2–3 orders of magnitude more active and its rate of formation is continuously changing?
- what role does homeostasis play in regulating nutrient function at the tissue level?

Each of these questions, which may be hypothetical but nonetheless including supporting evidence, is posed because too often they are ignored or superficially interpreted without biological context.

## The Biological Complexity of Nutrient Function

A discussion about the exceptional complexity of nutrient function would be well served to begin with the biological concepts of pleiotropy and epigenetics [4–7]. These concepts have been adopted by geneticists in order to go beyond the one gene—one enzyme hypothesis that started [7], then dominated the ‘genetic revolution’ for much of the past 75 years. Both concepts illustrate biological complexity. Geneticists define pleiotropy as “when one gene has an effect on multiple phenotypes” or, perhaps, “as multiple consequences of a single molecular function” [7]. While this hypothesis has greatly increased our understanding of post-genetic complexity, mutated genes continue to hold our attention in discussions on the development of cancer (and certain other diseases).

Epigenetics, which may be described as the terrain for this post-genetic complexity, still retains the feature of "heritable traits", but not necessarily those that are “attributable to sequence-specific changes in DNA”. More recently this discussion on epigenetics has been extended “to describe the study of chromatin biology” [8] and its ability to regulate “all DNA-templated processes, including transcription, repair and replication” [8, 9]. Although these concepts (the process of pleiotropy and the terrain of epigenetics) have produced a very large body of research literature, they still focus on gene-centric activities and their heritable traits.

The concept of pleiotropy, while broadening the scope of understanding gene action, also facilitates and expands our understanding of nutrient function as well, beyond the contemporary one nutrient-one mechanism-one outcome paradigm. A pleiotropy-like concept could infer, for example, the ability of one nutrient to upregulate or downregulate multiple gene-based mechanisms that lead to multiple phenotypes that converge on a common outcome. The following two examples are selected, from many others, to illustrate this pleiotropic-like effect. One represents a nutrient initiated health effect, the other a nutrient initiated disease effect.

Genistein is an extensively researched, estrogen-like isoflavone chemical in soy products, which exhibits anti-cancer properties [10, 11] and involves a large number of supporting mechanisms [12]. It blocks estrogen receptors, preferentially estrogen receptor-β [13], thus negating promotion of breast cancer by endogenous estrogen, it modulates genes that regulate cell cycling and programmed cell death (apoptosis), it inhibits the nuclear protein complex (nuclear factor-kappa-B) that activates DNA transcription responsible for stress factor induced cancer, it inhibits transcription and protein expression of prostate specific antigen that promotes prostate cancer and it protects cells against reactive oxygen species (ROS) that encourage cancer growth, among many other molecular events of cancer development. Aside from these many mechanisms affecting cancer development, some evidence also suggests that genistein lowers cardiovascular disease risk [14] and prevents osteoporosis [15]. In addition, there are other isoflavones and related components in soy and other legume products that undoubtedly play comparable perhaps synergistic roles with genistein to produce a spectrum of health benefits [16].

A second example arose from findings in my laboratory of a series of rodent experiments which investigated the effect of the feeding of animal-based protein on the development of liver cancer (hepatocellular carcinoma) initiated by aflatoxin, the most potent of all chemical carcinogens [17]. This research was conducted many years before the beginning of the human genome project thus could not use the more recent methodologies which identify and match specific genes with specific outcomes.

Preliminary (anecdotal) observations in the Philippines suggested that elevated protein consumption was associated with childhood liver cancer, at a time when it was assumed that this cancer was likely due to the consumption of aflatoxin [18]. At about that same time, Indian researchers showed that animal protein substantially increased aflatoxin-initiated tumor development in rodent studies [19]. Aflatoxin, like other initiating carcinogens, requires enzymatic activation, usually by the nutritionally modifiable [20] cytochrome P450 dependent, mixed function oxidase (MFO, later years named CYP450), mostly located in the liver. In these laboratory animal studies, aflatoxin was administered at a dose to maximize tumor formation whereas the modifying effects of protein (casein) were observed within a modest and relevant range of 5–20% of total diet calories.

Because animal protein promotion of liver cancer appeared to be so substantial, convincing and provocative, the responsible mechanism was sought. Higher dietary protein (20% of calories versus 5%) 1) increased MFO enzyme activity 3- to 4-fold [21–26], 2) increased MFO-catalyzed activation of aflatoxin [21] to 3) a highly reactive epoxide of aflatoxin that covalently bonds to DNA [27] and produce mutations [28, 29], 4) increased the number and/or size of pre-neoplastic cells in a dose-dependent manner (although this increase started at the recommended level of 10% protein) [30, 31], 5) diverted dietary energy (calories) away from its normal, healthy support of voluntary exercise [32] and basal metabolism 6) through increased thermogenesis and greater brown adipose tissue activity [33, 34] thereby favoring greater growth of cancer [35, 36], 7) increased IGF hormone production (observed in a companion liver cancer mouse model that was initiated by hepatitis B virus and promoted by protein) [37], 8) increased production of reactive oxygen species [38] known to promote cancer [39], 9) depressed natural killer cell activity that destroys cancer cells [40] and, ultimately, 10) increased mature tumor formation and early death [33, 41–43]. Also, increased dietary protein compromised DNA repair activity (unpublished). These disease-enhancing mechanisms appeared shortly after high protein feeding was commenced, often within 8–14 days but even as early as 24 hours [44]. Remarkably, dietary protein promotion of tumor development also regressed when dietary protein was decreased, the first time to my knowledge that cancer initiation by a powerful carcinogen could be turned on and off by relatively simple nutritional means [45–47]. During the several years of these experiments, the search for the responsible mechanism for enhanced tumor development by animal protein became ever more elusive. Each time a ‘key’ mechanism was sought, one was found.

Dietary protein completely controlled the ability of this very powerful carcinogen to produce tumors, both during early neoplasia (reviewed above) and in a two-year lifetime study [41, 43]. In this latter study, all 58 animals exposed to the carcinogen and given the 20% protein diet died with advanced tumors before two years. All 60 animals fed the 5% protein diet and provided the same high dose of carcinogen as animals on the 20% protein diet were alive, unusually thrifty and energetic, and free of cancer. And finally, this remarkable protein effect was produced by cow’s milk protein, casein—when fed in excess of the 10% level of dietary protein generally recommended to meet normal protein requirements [31]; wheat and soy protein did not promote this cancer, even at 20% dietary protein [41, 48]. The most remarkable finding of these studies was the turning on, then off, through two cycles of tumor development, by the feeding, respectively, of 20% then 5% protein diets.

Both of these examples illustrate a pleiotropic-like effect. One concerns disease prevention by a plant-derived chemical (genistein) while the other concerns disease promotion by an animal-derived chemical (animal protein). In each case, multiple mechanisms, likely linked to the expression of multiple genes caused by a single chemical/nutrient, participate in the development of the final outcome. Many more examples of other nutrients and nutrient-like chemicals can now be cited, especially those in recent years whose mechanisms of effect are being identified by the more specialized methodologies.

This discovery, 30–40 years ago, of a network of ‘explanatory’ mechanisms challenges the popular assumption of one nutrient (i.e., animal protein) affecting one gene (i.e., mutated by aflatoxin) that affects one disease (i.e., liver cancer). Multiple mechanisms from a single nutrient, each supporting the same outcome, illustrates a more comprehensive role for nutrition, one that acknowledges the health promoting and disease preventing effects of not one but multiple nutrients provided by whole food. Nutrients and their metabolites from whole plant-based foods produce an almost seamless stream of pro-health/anti-disease events. Each nutrient and its metabolites, depending on dose, engages multiple genes, each gene in turn expresses multiple enzymes that catalyze multiple reactions (phenotypes) that collectively converge to produce multiple disease and health outcomes (as an aside, the concept of ‘nutrition’ refers to the creation of health responses while ‘malnutrition’ refers to the creation of disease responses).

It is imperative that a description of nutrition recognize the under emphasized but fundamental property of biological complexity, which may be demonstrated at all levels of biology, from whole body physiology to subatomic particles. Consider, for example, the biological level of a single enzyme which, in its simplest form, catalyzes the conversion of a single chemical (substrate) into a specific product. The mixed-function oxidase [49], also known as the drug metabolizing enzyme, once thought to primarily exist in the liver is now known to exist in many other tissues as well. This ‘single’ enzyme, which is a complex of electron transfer- and heme-containing cytochrome proteins within a phospholipid matrix, displays an awesome breadth of enzymatic activities. There are 57 known cytochromes available for use by this enzyme and seemingly infinite configurations and combinations of these proteins and phospholipids enabling it to oxidize a variety of organic chemicals. Some enzyme variants participate in synthesizing endogenous metabolites (steroids, fatty acids), some oxidize chemicals foreign to the body (environmental and pharmaceutically active chemicals) to prepare them for their excretion from the body and some variants activate foreign chemicals generally as a first step to detoxification and excretion (carcinogens and certain pharmaceuticals). The many forms of this enzyme are referred to as a superfamily of monooxygenases that are capable of catalyzing an incredible variety of substrate structures. Further, a wide variety of chemicals and nutrients [20] can substantially modify this enzyme activity, either by inducing protein synthesis [50] or by rearranging its molecular configuration [51]. The versatility of this enzyme is especially impressive in its ability to simultaneously activate and de-activate substrates like carcinogens, an exceptional juggling act. For this supposedly ‘single’ enzyme, there are so many ways for it to customize the metabolism of a wide variety of chemical structures, some of which chemicals may never have been seen before by this enzyme.

These observations on nutrient function extend beyond pleiotropy that, for geneticists, continue to assume genetic causality and simultaneous retention of hereditable traits. For nutrition, I suggest a new term, epitropy, which greatly expands the scope of pleiotropy and, unlike pleiotropy, does not retain heritable traits. Aside from the assumption of epitropy which hypothesizes that each nutrient and/or its metabolite(s) influences the expression of multiple genes, via multiple mechanisms, to yield multiple enzyme products and related outcomes, still more complexity exists. That is, any assumption of mechanisms of action for a single nutrient at a single moment in time will change, being modified by time and other nutrients that are simultaneously consumed in food.

Nutrients in known health promoting foods likely act cooperatively to produce the food’s benefits, illustrating the concept that the whole is greater than the sum of its parts. For example, plant-based foods contain many antioxidants, some being water soluble (vitamin C and various phytochemicals) and some being oil soluble (e.g., β-carotene and other carotenoids, multiple vitamin K analogs). Both antioxidant types minimize exposure to excessive free radicals that cause tissue damage and disease formation. Interception of free radical formation by both types of antioxidants would mutually benefit the other antioxidant group, thus producing an effect greater than the sum of its parts. Even a higher order of complexity exists because each taxonomic group of plants will have multiple plant foods, each with its own unique collection of nutrients that may repress and even reverse disease formation, perhaps even in a complimentary way. Some of these plant types provide energy, some the antioxidants, some the right kind and amount of dietary fat, some the vitamins, some the complex carbohydrates and some the right combination of minerals. Perhaps the most remarkable property of these plants and their nutrients is their ability to work in concert to produce the same outcome.

An awareness of the infinite complexity of nutrient function, especially when including the additional impact of nutrient-nutrient interactions that shift within micro-units of time, helps to explain why nutrients behave differently when consumed alone (e.g., supplements) or when consumed as part of the whole food. Comprehensive reviews over the past two decades, for example, have mostly concluded that vitamin supplementation has little or no supporting evidence [52–59], much to the chagrin of the huge industry that survives on these claims. The wholeness of food illustrates how nutrients, working collectively and dynamically, create health.

## Influence of Dietary Choice on Nutrient Function

In an odd sort of way, another property that helps to interpret nutrient function within a total diet context is the effect of food choice. There are many reasons why people choose the foods they eat, mainly related to social, economic and convenience motivations [60–63]. Choosing food according to its nutrient content, as reflected by shoppers who read nutrient labels, has become more significant in recent decades. But for a longer period of time, even more than a century ago, the importance of consuming protein, especially protein of ‘high quality’, has been a pervasive desire, either consciously or unconsciously, ever since its discovery in 1839 [64]. This mainly meant a desire for animal based food, because it was first thought that protein was present only in animal-based foods, a lingering belief even today for many people. But this preference for protein has biological consequences, especially because consuming more protein-rich, animal-based foods modifies the nutrient composition of the rest of the diet, thus setting up an important biological and nutritional contrast of animal versus plant-based foods. The nutrient contents of sample blends of animal- and plant-based foods, along with highly processed foods, is shown in Table 1 [65, 66]. Assuming a constant daily calorie intake, exchanging plant based foods for animal-based foods in order to consume the much-desired animal based protein, will cause substantial changes in the consumption of other nutrients. Differences in nutrient composition for animal and plant-based foods of 10-fold or more exist for seven of the listed nutrient entries.

Also in recent years, more diet and health researchers are turning their attention to the comprehensiveness of the nutritional effect by investigating associations of disease risk with dietary patterns of foods and nutrients. This suggests a growing awareness of the broad effect of whole food on health and disease outcomes, an awareness that is consistent with the epitropic and wholistic characteristics of nutrition function at the cellular and physiological levels presented here. During 2016 and early 2017, an unusually large number of research publications are focused on dietary patterns, which have been creating a consensus that meat and other animal based foods increase while whole plant-based foods decrease risk for a broad variety of chronic disease outcomes, including overall cancer occurrence [67], cancer survivorship [68], type 2 diabetes [69], bone mineral density [70, 71], lung function [72], age-related macular degeneration [73], cardio-metabolic and endocrine biomarkers [74], hip fracture [75], offspring adiposity [76], mental depression [77] and diverticulitis [78], among many others.

In brief, this evidence on food patterns shows an unusual breadth of outcomes consistently associated with higher disease risks for animal-based foods and/or lower disease risks for plant-based foods. Taking into consideration the multiplicity of nutrient inputs, the multiplicity of mechanisms and the multiplicity of outcomes considered earlier, the cause and effect relationship between diet and health or disease becomes more convincing and less encumbered with the inevitable controversies that arise when considering out of context the individual contributions of specific nutrients and other risk factors.

One of the most convincing and substantive findings for a total diet effect is that of the Esselstyn research group [79], who used the whole food plant-based diet in an intervention study to show that it almost completely reversed coronary heart disease. Among 198 consecutive patients counseled to use this dietary lifestyle, only one individual among the 177 adherent participants (<1%) suffered a recurrent event (stroke) within the next 3.7 years. Among the 21 non-adherent participants, 13 (62%) suffered recurrent events. This affirmed an earlier smaller study of 18 patients by the Esselstyn group [80]. During the next 25 years, no one in this latter, smaller cohort suffered a recurrent event, including 5 patients before they died from non-cardiac events [81]. Similar results were earlier reported by Ornish et al., with findings published after one [82] and five years [83] of treatment. The study by Ornish et al., who also included stress management and exercise in addition to diet [82], was the first to demonstrate with peer-reviewed findings this whole food plant-based effect. The ability of whole food, plant-based nutrition to control cardiovascular disease is far superior to any pills-and-procedures medical intervention ever undertaken on this disease [79, 82], as also indicated for prostate cancer treatment [84].

These impressive short-term, diet-dependent effects on disease risk are consistent with similar long-term effects in population studies. Both findings undoubtedly include direct effects of animal protein and, perhaps even more importantly, the indirect effects caused by decreased consumption of plant based foods. This was reported in 1959 by Jolliffe and Archer [85] who compared mortality rates for a broad collection of cardiovascular diseases among 20 countries and again in 1986 by Carroll et al. [86] who compared mortality rates for breast cancer among 38 countries. The regression of disease mortality on dietary animal protein passes through the X:Y origin for both diseases, suggesting that there is little or no theoretically ‘safe’ level of animal food consumption. The association of breast cancer with saturated fat (much more common in animal based foods) that was initially published appears to have been an anomaly because prior evidence in laboratory animal studies showed that it was more likely polyunsaturated fat, not saturated fat, that stimulates tumor promotion [87, 88], a distinction that occurs only when total dietary fat is high. Therefore, the saturated fat correlation observed for this international study is therefore likely to be much better explained by its very close correlation with dietary animal-based protein [89, 90].

## Nutritional Reasoning

These varied types of evidence concerning food and health associations—observational, intervention and mechanistic, when combined with wholistic, epitropic interpretation of nutritional function, supports the hypothesis that a whole food plant-based dietary lifestyle is the most comprehensive and profound means by which food favors human health, reversing heart disease, turning experimental cancer on and off, and demonstrating a considerable breadth and rapidity of effect for a variety of illnesses [65, 66].

This hypothesis should have far-reaching consequences. The most significant may be the development of dietary guidelines and related public health policy positions. For example, constructing U.S. dietary guidelines starts with the establishment of requirements and recommendations (RDAs) for individual nutrients. First published in 1941 [91], these recommendations have been updated by experts every five years since. This exercise—beginning with nutrients and ending with a message on foods and food patterns— often creates raucous debate and controversy each time a new version of the dietary guidelines is published. Except for an ever-expanding body of experimental details, the message for the latest (and eighth) guidelines for 2015–2020 is not very different from the guidelines first published in 1980. Detailed research findings, often contradictory, have greatly increased but it is not clear that this information has led to advances in public health policy on diet and health policy.

I propose that the reason for this unsatisfactory ‘progress’ can largely be traced to a major misunderstanding of nutritional science and how it is investigated and applied. First, we rely on an assumption that nutrients act rather independently, as inferred by investigating specific activities of individual nutrients in controlled laboratory experiments or by adjusting nutrient correlations with disease outcomes in epidemiological studies for confounding variables. This assumption of nutrient independence, in reality, is superficial because nutrient activities depend on which function is chosen for study and, often, on relatively narrow experimental contexts which may substantially vary among studies. It ignores the ever-changing kinetic behavior of nutritional function and a range of possible outcomes, many of which may not yet be known.

Understanding nutrition fell victim to the 19th century idea that cancer was a “local” disease, meaning that it could be surgically removed [92]. There was no need to consider the then-existing alternative hypothesis that cancer was a “constitutional” disease (that is, whole body involvement), which would have implied a role for nutrition. A serious proposal to test the effect of a vegetable diet on cancer patients, for example, was twice proposed then rejected at the famed Middlesex Hospital in London in 1809 [93] and 1815 [94]. For the remainder of the 19^th^ century [92], the local theory of disease gained ascendancy, becoming a tap root for the development of modern day reductionist theory of disease and the use of single agents, like chemotherapy and other drugs to treat disease. Nutrition, unless a causal nutrient could be identified, was not to be taken seriously [92].

Historically, a second flaw followed from the first. It mostly relies on inductive reasoning commonly used to assess what type of overall dietary practice optimizes health and prevents disease. In the development of dietary guidelines, for example, information flows from recommendations of individual nutrient intakes to their eventual use for constructing whole food recommendations. This strategy invites bias because it is possible to select recommendations on nutrient amount and type that favors a pre-conceived optimum diet. For example, consider the possible bias that can arise from underestimated recommended daily allowances (RDAs) for vitamin C (75–90 mg/day, compared with 300–500 mg/day generally available in a healthy whole food), overestimated RDAs for protein (from 8–10% of total calorie intake to an astounding 35% of calories as the ‘tolerable upper limit’ [95]) and calcium (from 500–700 mg/day in countries with low risks of osteoporosis to 1000–1300 mg/day in countries with high risks of osteoporosis [96]). Collectively, these biased recommendations substantially favor the consumption of more animal-based foods and less plant-based foods.

In contrast, deductive reasoning begins with reliable ‘big picture’ evidence on the association of food consumption with human health and disease. For the past several decades, this mostly came from studies that compared food consumption with health/disease outcomes among countries and by prospectively following cohorts of a large number of individuals. Ideally, dietary experience should be as broad as possible, in order to assess the effect of nutrition at its limits. International studies with ethnically diverse populations provide the greatest dietary range while cohort studies usually have a more limited dietary range because they include individuals from the same population (almost none of these studies have included a significant number of participants using a whole food plant based diet). These studies provide information on food intakes, from which nutrient intakes can be estimated. The associations, or correlations, of nutrient and food intakes with health/disease outcomes are then assessed for statistical significance, determined either at fixed times (‘cross-sectional’) or, if possible, as time-dependent trends. These correlations have mostly focused on individual foods and nutrients as possible causes of disease, being mindful of the limitation that correlations do not infer causation.

During the last couple of decades, however, attention has turned to an analysis of the effects of dietary patterns (instead of individual foods and nutrients) on disease outcomes [97], which is a welcome new direction that is more consistent with the wholistic interpretation of nutrient function considered here. Seeking dietary pattern associations with disease mortality rates was a major objective of our diet and disease study in rural China [98–101]. The advantages and disadvantages of using dietary pattern analysis are summarized elsewhere [97]. Although it is assumed that nutrient composites explain the nutritional effect on disease outcomes better than individual nutrients, it is said that one ‘disadvantage’ of the dietary pattern (nutrient composite) analysis is its minimizing the ability to detect significant health effects of individual nutrients [97]. This is a fair criticism if identification of individual nutrient causation of disease is the goal. But, except for rare circumstances, this should not be the goal. The goal should be to identify the whole food based nutritional experience which 1) minimizes risk for the broadest array of illness and disease, 2) simultaneously treats and prevents illness and disease, 3) shows that nutrition of whole food is superior to the so-called nutrition of individual nutrients of that food, 4) minimizes the need for pharmaceutical chemicals to restore health and 5) promotes a longer, disease-free life, while 6) simultaneously promoting other health related socioeconomic benefits. I submit that the WFPB dietary lifestyle without added oil, salt and refined carbohydrates approaches this “ideal” diet far better than any other concoction of pills and procedures and of fragmented food parts of the whole.

Returning to the proposal to use deductive reasoning for food and health policy development, let’s start with an hypothesis on the superior health benefits of the WFPB dietary lifestyle, which hypothesis can be falsified (a critical criterion for scientific proof of concept). The next step in this exercise is to assess and describe the profile of nutrients that best typifies this ideal diet. And finally, determine the biologically plausible mechanisms used by those levels of nutrient intake that consistently and simultaneously support broad-based health maintenance and disease remediation (treatment). This is a top down analysis that begins with evidence believed to support the broadest scope of nutritional benefits (described above). There is, for example, consistent and virtually indisputable evidence obtained in population and cohort studies showing that higher consumption of various types of dietary fiber (complex carbohydrates) and dozens of antioxidants along with lower consumption of added oils/fats and animal-based protein are each associated with lower rates of many types of cancer, cardiovascular diseases and some autoimmune diseases, as summarized in many institutional, expert committee and meta-analysis reports reported earlier in this paper. This type of evidence enjoys broad support from disparate groups, is most resistant to research bias and consistently points to the health value of a diet rich in whole plant-based foods. Starting from this hypothetical ‘big picture’ base of information (or any other testable hypothesis), we then explore biological plausibility and consistency of activities of the nutrients that typify this dietary pattern. This is deductive analysis and the evidence derived therefrom clearly shows that a diet of whole plant-based foods produces the most convincing evidence. In contrast, inductive analysis, which is commonly used to create dietary guidelines and other types of general information, would tend to select activities of isolated nutrients studied out of their living context in order to create broad-based dietary recommendations.

## Human Nutrition and Mother Nature

When I speak of nutrition, the evidence that I see emerging appears to be what Nature has constructed for us over the eons of time of our existence. Unfortunately, we think of foods as collections of individual nutrients, perhaps acting independently. But this interpretation strays from the natural order of things. One of the best examples of this misinterpretation is the differing responses produced by nutrients consumed in pill form when compared to the same nutrients consumed in food form. When consumed in isolation—as in pills or supplements—nutrients are not being subjected to the modifying effects of other factors in whole foods. Exacerbating this problem is the ‘dose’ of isolated nutrients being consumed, amounts that are likely to be far from the natural, food-based customs.

A major reason why isolated nutrients, as supplements, are still used by the public is the false assumption (or excuse?) that we can retain the desired health while continuing to eat our favorite foods, however unhealthy they may be. It is a message widely promoted, at least inferred, by the nutrient supplement industry who also make highly questionable health claims. Another reason pills are chosen instead of food is the difficulty of acceptance that many people perceive (and sometimes experience) when switching their diet from animal-based and convenience foods to whole plant-based foods. This perception, highlighted by the industry, is unfortunate because it is the nutrition of whole plant-based foods, not isolated nutrients, that produces the extraordinary health benefits discussed here. We are addicted to our present diets, specifically addicted to the high fat, sugar and salt contents but, like most other addictions, these can be resolved. It may take a month or two but it can—and does—happen in most cases until a new health reality is experienced, the one that Nature fashioned for us.

Still, the health benefits associated with whole plant-based foods are not yet sufficiently proven for many people. Thus I suggest that the evidence supporting the health value of a whole food diet be considered an hypothesis, not a proven fact. For me, this evidence is more than convincing enough to make major decisions, public and private. Whether this evidence rises to the level of a traditionally defined ‘fact’, is not necessarily the right question. Very little traditionally produced biomedical research evidence ever becomes unequivocal fact because what may be true for one set of experimental conditions may be different for other conditions. For example, in large datasets, a small number of so-called outliers are not uncommon. A major reason for these outliers—other than those attributable to methodological errors—is often due to the narrow scope of the hypothesis being investigated, a classic sign of scientific reductionism. The narrower the scope of diets being investigated, the more compromised becomes the evidence supporting food guidelines for the larger population.

It also should be noted, regrettably, that among diet and health studies on large cohorts of people, virtually none of these cohorts, to date, have included individuals accustomed to using the whole food plant-based dietary lifestyle. Vegans and vegetarians approach the nutrition of this dietary lifestyle and, in doing so, are known to have lower mortality rates for non-communicable chronic diseases [102, 103] but this cannot be the full expression of health effects because, according to the most robust of dietary studies on vegans and vegetarians, the mean contents of total fat and sugar for vegans, vegetarians and meat eaters were the same, 30–31% fat and 22–23% sugar [102]). The absence of individuals customarily using the whole food plant-based diet in these large cohorts is due, in some considerable part, to the long-standing custom of professional and governmental authorities and institutions to proactively minimize, at least question, the validity of information on the exceptional nutritional value of plant-based diets for the public. Many people consider this information to be relatively new and not sufficiently tested.

An ideal experiment on the health benefits of a whole food plant based diet should include the full range of dietary experience. It would be a massive undertaking, probably involving a treatment group of hundreds of thousands even millions of individuals, a large portion of their lifetime, and a reliable monitoring of food intakes and health outcomes; a comparable control group using some version of the contemporary diet would also be needed. Such a study will never happen. Short of this ideal, investigating such broad, complex hypotheses will depend on comparing the results of varied but more narrowly focused experimental study designs, each offering a different perspective, with the results of such studies examined for their internal consistency. Guidelines for such investigations are available, probably the most reliable being those of Sir Bradford Hill [104]. Over time, the evidence from these various studies gradually accumulates, to a time when the evidence is sufficiently reliable for decision making, either by individual and/or by institutions.

In spite of these limitations of classical experimental research, there still is an abundance of evidence which supports, as a goal, the use of a diet wholly composed of whole food plant-based foods, without added oil and refined carbohydrates. Two kinds of evidence convincingly exposes the need for dietary change and offers a solution to support that change. These are 1) breadth of effect [66] and 2) reversal of disease [79, 82, 105] Breadth of effect evidence challenges the contemporary and popular concept of ‘targeted drug therapy’ which encourages research and development of specific drugs for controlling specific diseases, a pharmaceutical market bonanza for the future. Reversal of disease means using the same nutritional protocol to prevent and to treat disease which challenges the use of pills and procedures to control disease. Both of these kinds of evidence are best appreciated when considering and appreciating the infinitely complex, dynamic and unknowable details of nutritional function.

The number of chemical (nutrient) entities in food that participates in nutritional function is infinitely large and mostly unknowable. The proportion of nutrients passing through each stage of digestion, intestinal absorption and metabolism is incalculable and ever changing—it is an infinitely dynamic system. Nothing is static. I would add two more impressive properties of the dynamics of whole body nutritional function, organ-to-organ communication across relatively vast distances within the body (a space feature) and replication of these properties through a very large number of cell generations during a lifetime (a time feature). No adjectives can adequately describe the awesome dynamics of the living biological system. Ideal nutrition, as produced by the whole food plant-based diet, supports each of these features and this is the rationale for understanding nutrition as a wholistic function.

We also know that this unknowable complexity is highly integrated and managed—homeostasis comes to mind. This is an awesome display of Nature, begging the question of its roots and its purpose. My best but very simple guess, mechanistically speaking, is that the body possesses an infinite wisdom at any point in time to know which nutrients, nutrient metabolites and related food substances to use for each of its wide diversity of needs, at every nanosecond in time within the trillions of cells in our body. This is the essence of nutrition—utilizing external food resources endowed with energy captured from the sun in the form of carbon-hydrogen bonds, extracting the energy of these bonds (e.g., ATP), then sending nutrients and their metabolites through carefully choreographed and infinitely complex systems to create life. This is the essence of Nature! No prescription of pills and procedures can ever mimic or surpass this wonder of Nature.

## Fulfilling the Potential of Nutritional Science

One last question: why is the science of nutrition, which I have found to be an unusually scholarly discipline and an effective prescription for health, not taught in medical schools, not deserving of a dedicated research institute at NIH and not deserving of a dedicated medical specialty? Why also, is the science of nutrition taught in academic nutrition departments so lacking in critical thinking and practical meaning? Also, within the fierce debate on the merits and demerits of the Affordable Care Act (i.e., Obamacare), why are both parties to the debate failing to consider nutrition as a means to reduce health care costs and a means to find accommodation? The present debate mostly focuses on who will pay the future bill, as if this debate is only about wealth, not health. Public information on nutrition is meager, entirely unseen or seriously distorted. If properly presented to the public, I am confident that a large number of people would listen, learn and opt for taking responsibility for their own health. In so doing, they would discover its surprising health benefits, realize huge savings in health care costs, better understand its contribution to animal welfare and recognize the effect of food choice on perhaps the most ominous problem of all, the frightening degradation of our environment. But most people do not choose this option because it is not available. Contemporary public health advisories, too closely tethered to corporate interests, are minimizing or even silencing this nutrition information, intentionally or unintentionally. Having been personally and deeply involved in these advisory panels, I contend that we too often fear compromising the food and drug marketplace. Very simply, we lack courage. This is a multi-faceted problem of personal and public health that can be resolved by understanding the science of nutrition.

## Figures and Tables

**Table 1:** Nutrition composition of three diet types.

Nutrient Differences			
	Whole Plants	Animal Foods	Refined Plants
Protein (g)	29	51	6.5
Lipid (g)	6	34	21
Carbohydrate (g)	97	8.6	72
Fiber (g)	27	0	1.8
Calcium (mg)	410	250	31
Iron (mg)	8.4	3.5	0.9
Potassium (mg)	2,600	1,200	350
Vitamin C (mg)	440	0	4.3
Folate (mcg)	640	64	15
B12 (mcg)	0	5.2	0
Vitamin A (IU)	25,000	680	18
Cholesterol (mg)	0	410	0

Whole plant blend: 100 kcal each of mango, pea, broccoli, kale, oats

Animal food blend: 100 kcal each of whole milk, chicken, beef, salmon, egg

Unenriched refined plant blend: Potato chips, spaghetti, cola, doughnut, Italian dressing
